# Supplementary material for: Patterns of Intron Gain and Loss in Fungi
Source: PLoS Biol. 2004 Nov 30;2(12):e422. doi: 10.1371/journal.pbio.0020422 (PMC532390; doi:10.1371/journal.pbio.0020422)
Supplement: Table S1 — Also available at http://genes.mit.edu/NielsenEtAl/. (4.3 MB ZIP). [file pbio.0020422.st001.zip › NielsenEtAl/html/804.html]

AN0050.1.NCU06417.1.MG06488.1.FG07302.1


```
 CLUSTAL W (1.82) Multiple Sequence Alignments - Introns Inserted


Sequence 1: MG06488.1	383 aa
Sequence 2: FG07302.1	378 aa
Sequence 3: NCU06417.1	394 aa
Sequence 4: AN0050.1	381 aa
Alignment Length: 414 aa
Number Identitical Residues: 173 aa
Alignment Score (without introns) 8721


MG06488.1 	-MASSNRIVDIHTHMYPPSYVDILKSRQTIPLVRTFDPAQDPRLILLAAEVESLNNAASS
NCU06417.1	MGFGDGKVVDIHTHMYPPEYVKILESRTSIPLVRSFPGSPDPRLILLDTEVPILEEAEAA
FG07302.1 	MSSSSARVVDIHTHMYPPSYIQILESRSAIPLVRKFPQASDPRLILLEAEVKALEEATR-
AN0050.1  	----MPLIVDVHTHVYPPAYMQMLRSRKTVPYVHDPSNNRDPRLIILSSD----DDAS--
          	       :**:***:*** *:.:*.** ::* *:      *****:* ::    ::*   

MG06488.1 	DAP-DKRANPPGRPLTKHYASLGQKIHFMNTHKIDISVISLANPWLDFLDPSEAGSIGES
NCU06417.1	KARGETPANIPGRPLTKHYSSLNQKMHFMETHEIDISVVSLANPWLDFLSASEAGPIAES
FG07302.1 	----DSEAKPPGRPLTSHYASLAQKVHFMDTHKIDISVISLANPWLDFLDPSESGSIAES
AN0050.1  	-----IPLDQRGRPVDSSYWDINVKLSFMRQHGINCSVISLANPWLDFVEPAEAQMWAER
          	        .  ***: . * .:  *: **  * *: **:*********:..:*:   .* 

MG06488.1 	INNEFDSMCN-----QHPG---------RLFFFATLPLTAP-LDTILACISHVKSLKYCR
NCU06417.1	INADFSRMCE-----EKCG---------RLFFFAALPLTAP-RDVILASIAHVSNLPYCR
FG07302.1 	VNQEFSRMCG-----EHPG---------RLFFFGTLPLTAN-LETILESIKSLPKLKYCR
AN0050.1  	INDDLEKTCATVNKAADPGNTLTLDQKETLFAFGALPLSAPSSESVVAEIKRLKTLEHLR
          	:* ::.  * : ..: . *.: : .... ** *.:***:* : : ::  *  : .* : *

MG06488.1 	GVILGTSGLGKGLDDPELLPIFEALGAARLMVFLHPHYGLPNDVWG-PRASAEYGHVLPL
NCU06417.1	GVILGTSGLGKGLDDPDLLPVFHALADAKLMIFLHPHYGLPNEVWG-PRAKENYGHVLPL
FG07302.1 	GVILGTSGLGKGLDDPELLPIFEALARAELTVFLHPHYGLPNDVWG-PRASAEYGHVLPL
AN0050.1  	GVIMGTSGLGKGLDDARLDPVWEALQETDMLMFLHPHYGLPEEAYGGPETTGRYGHVLPL
          	***:***********. * *::.**  : : :*********::.:*.*.:. .*******

MG06488.1 	ALGFPMETTIAVARMYLAGVFDRVPSLNMLLAHSGGTLPFLAGRIESCILHDGHLMREGK
NCU06417.1	ALGFPMETTIAVTRMYLAGVFDQVPKLNMLLAHSGGTLPFLAGRIESCILHDGHLHSAAG
FG07302.1 	ALGFPMETTIAVARMYLAGVFDKVPDLRMILAHSGGTLPFLAGRIESCIMHDGQFLREGK
AN0050.1  	ALGFPLETTIAVTRMLLSGVFDRFPRLKILLAHSGGTLPFLAGRIESCILHERKFISGGG
          	*****:******:** *:****:.* *.::*******************:*: ::   . 

MG06488.1 	AGGPGSGRRTIWDVLKDQVYLDAVIYSEVGLKAAIDASG---VDRLMFG~TDHPFFPPLT
NCU06417.1	TKP----KKTIWEVLSSQIYLDAVVYSDVGLKAAVQASGPEGHERLMFG~TDHPFFPPLG
FG07302.1 	LAK---GRRTVWDVLKEQIYLDAVIYSEVGLKAAIQASG---ADRLMFG~TDHPFFPPLT
AN0050.1  	DVQ--GPQRSVWDVLKTNIYLDAVVYGKPGLEAAMTASG---SDRLLFG1TDHPFFPPLD
          	     . ::::*:**. ::*****:*.. **:**: ***    :**:** ********* 

MG06488.1 	SDEDGAWESVGLNAEAVDKALGSGST-------AAAGVMGENAIRVLRLYES---
NCU06417.1	SDEEGEWESVTWNGAAVRKAFGAEDGEDSEEGKKVRGVMGANAVRVLNLRRDGCR
FG07302.1 	SDEQGEWESVSLNAEAVIRAVGQGTG-------DCENVMGMNAVKILRLDSES--
AN0050.1  	S-KDNSWPSVTTNYQAIHATFDTNSK-------TVADVLGGNAARILNLK-----
          	* ::. * **  *  *:  :..              .*:* ** ::*.*
```
